# Supplementary material for: Development and Optimization of Alpha-Pinene-Loaded Solid Lipid Nanoparticles (SLN) Using Experimental Factorial Design and Dispersion Analysis
Source: Molecules. 2019 Jul 24;24(15):2683. doi: 10.3390/molecules24152683 (PMC6696006; doi:10.3390/molecules24152683)
Supplement: Supplementary file 1 [file molecules-24-02683-s001.pdf]

Supplementary Material

Table S1. Analysis of A) the mean particle size (Z-Ave), B) the polydispersity index (PDI) and C) zeta potential (ZP) by ANOVA statistical test.

A)

Evaluated factors and their interactions	Sum of squares	Degrees of freedom	Mean square	F-value	p-value
**(1)Imwitor® 900 K**	1984717	1	1984717	6.173713	0.088900
**(2)Poloxamer 188**	2062096	1	2062096	6.414409	0.085225
**1 by 2**	1909648	1	1909648	5.940199	0.092727
**Error**	964436	3	321479		
**Total**	6920897	6			

ANOVA; Var.: Z-Average; R-sqr=.86065; Adj:.7213 (FD_Alpha-pinene (1).sta) 2**(2-0) design; MS Residual=321478.7 DV: Z-Average.

B)

Evaluated factors and their interactions	Sum of squares	Degrees of freedom	Mean square	F-value	p-value
**(1)Imwitor® 900 K**	0.063504	1	0.063504	2.779458	0.194071
**(2)Poloxamer188**	0.066049	1	0.066049	2.890848	0.187643
**1 by 2**	0.034225	1	0.034225	1.497968	0.308339
**Error**	0.068543	3	0.022848		
**Total**	0.232321	6			

ANOVA; Var.:PDI; R-sqr=.70496; Adj:.40993 (FD_Alpha-pinene (1).sta) 2**(2-0) design; MS Residual=.0228476 DV: PDI

C)

Evaluated factors and their interactions	Sum of squares	Degrees of freedom	Mean square	F-value	p-value
**(1)Imwitor® 900 K**	0.000784	1	0.000784	0.078011	0.798159
**(2)Poloxamer 188**	0.005329	1	0.005329	0.530259	0.519189
**1 by 2**	0.001369	1	0.001369	0.136221	0.736577
**Error**	0.030149	3	0.01005		
**Total**	0.037631	6			

ANOVA; Var.:ZetaPotential; R-sqr=.19882; Adj:0. (FD_Alpha-pinene (1).sta) 2**(2-0) design; MS Residual=.0100498 DV: Zeta Potential

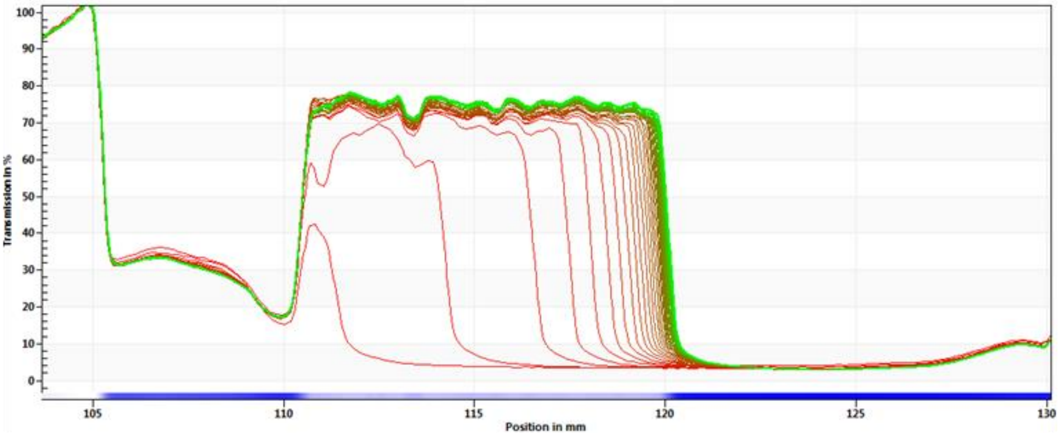

A) SLN1, 25ºC - No observable separation.

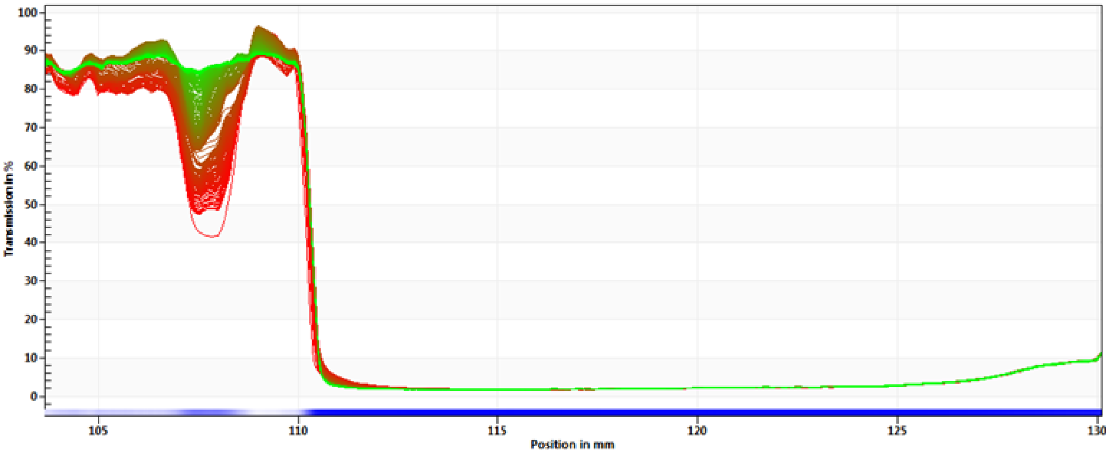

B) SLN2, 25ºC - No observable separation. Differences occur before the meniscus.

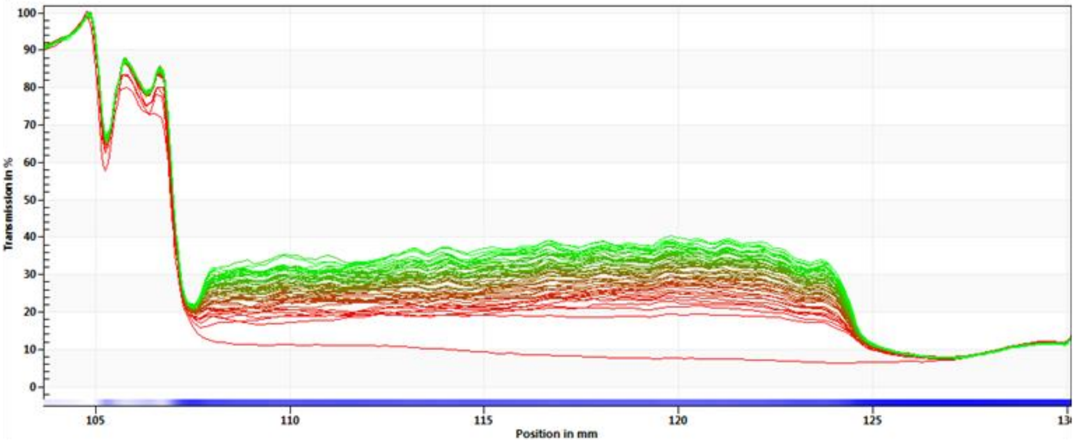

C) SLN3, 25ºC - Separation process: sedimentation.

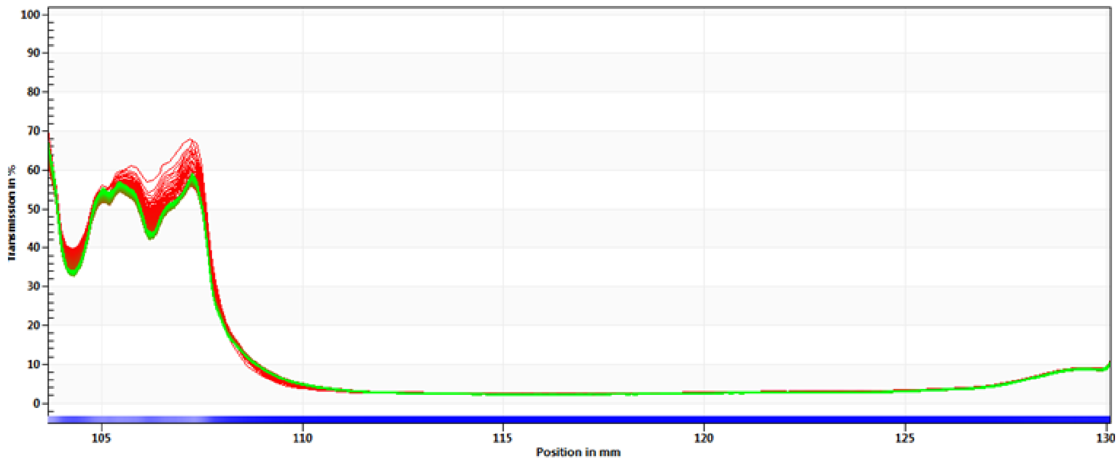

D) SLN4, 25ºC - No observable separation.

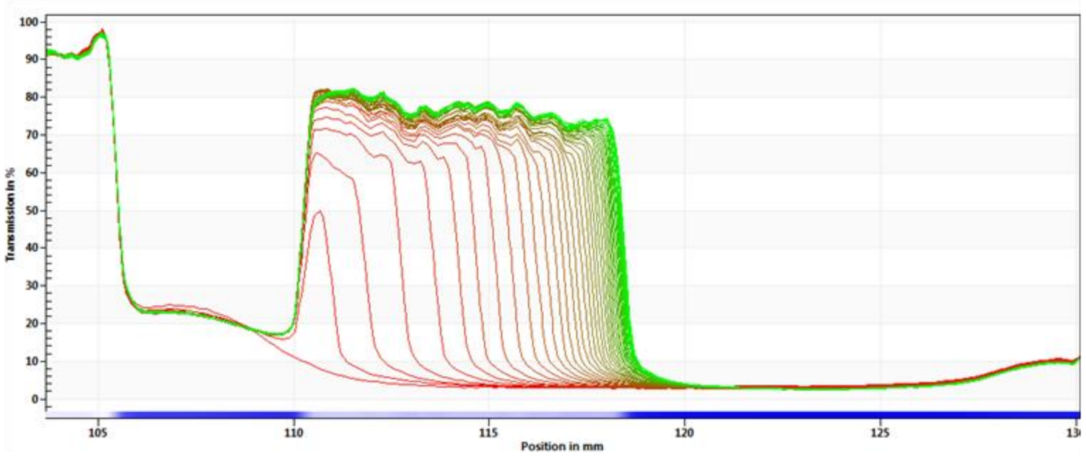

E) SLN6, 25ºC - Separation process: sedimentation with particle compaction.

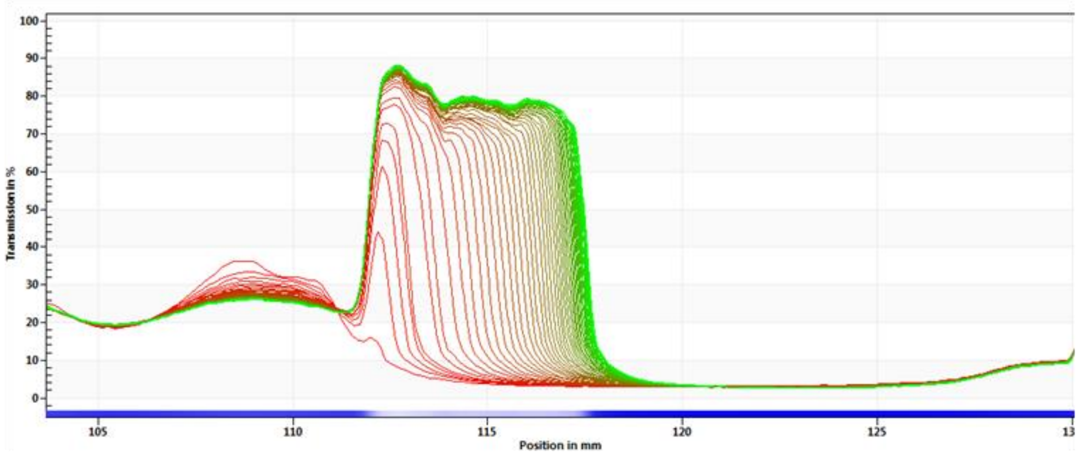

F) SLN7 25ºC - Separation process: sedimentation with particle compaction.

Figure S1: A) SLN1, 25°C - Separation process: sedimentation with particle compaction; B) SLN2, 25° C - No observable separation. Differences occur before the meniscus; C) SLN3, 25°C - Separation process: sedimentation.; D) SLN4, 25ºC - No observable separation; E) SLN6, 25°C - Separation process:

sedimentation with particle compaction; F) SLN7, 25°C - Separation process: sedimentation with particle compaction.
